# Advanced oxidation protein products induce Paneth cells defects by endoplasmic reticulum stress in Crohn's disease

**DOI:** 10.1016/j.isci.2023.107312

**Published:** 2023-07-10

**Authors:** Jie Shi, Weidong Wang, Shibo Sun, Xiaoping Xu, Jieying Fei, Qian Zhou, Caolitao Qin, Shiyu Ou, Fengfei Wu, Fang ting Wu, Tianyan Xu, Lan Bai, Fang Xie

**Affiliations:** 1 Guangdong Provincial Key Laboratory of Gastroenterology, Department of Gastroenterology, Nanfang Hospital, Southern Medical University, Guangzhou, Guangdong 510515, China; 2 Department of Radiation Oncology, The Sixth Affiliated Hospital, Sun Yat-sen University, Guangzhou, Guangdong 510655, China; 3 Biomedical Innovation Center, The Sixth Affiliated Hospital, Sun Yat-sen University, Guangzhou, Guangdong 510655, China; 4 Department of Hepatobiliary Surgery, Nanfang Hospital, Southern Medical University, Guangzhou, Guangdong 510515, China; 5 Department of Gastroenterology, Hunan Provincial People’s Hospital, Changsha, Hunan 410005, China

**Keywords:** Cell biology

## Abstract

Paneth cells (PC) play a key role in the innate immune response of intestine epithelium, and PC defects contribute to the pathogenesis of Crohn’s disease (CD). In this study, we utilized active CD tissues and advanced oxidation protein products (AOPP)-challenged C57BL/6 mouse model to investigate the effect of AOPP on PC defects in CD. We found that AOPP accumulated in active CD tissues and was negatively associated with lysozyme expression, while positively correlated with the presence of ER stress markers. Furthermore, AOPP treatment induced PC defects mainly through excessive ER stress *in vivo*, and AOPP also caused mitochondria-associated ER membranes formation and mitochondrial dysfunction. In addition, the effects of AOPP could be attenuated by the administration of ER stress inhibitor, TUDCA. These findings suggest a pathogenic role of AOPP contributing to PC defects and may provide the basis for developing new strategies to managing CD.

## Highlights

• In active CD tissue, AOPP accumulation associated with PC defects and ER stress
• AOPP challenge resulted in PC defects and ER stress in mice
• TUDCA, an ER stress inhibitor, effectively mitigated AOPP-mediated PC defects
• TUDCA alleviated AOPP-induced MAM formation and mitochondrial dysfunction

## Graphical abstract

**Figure:**
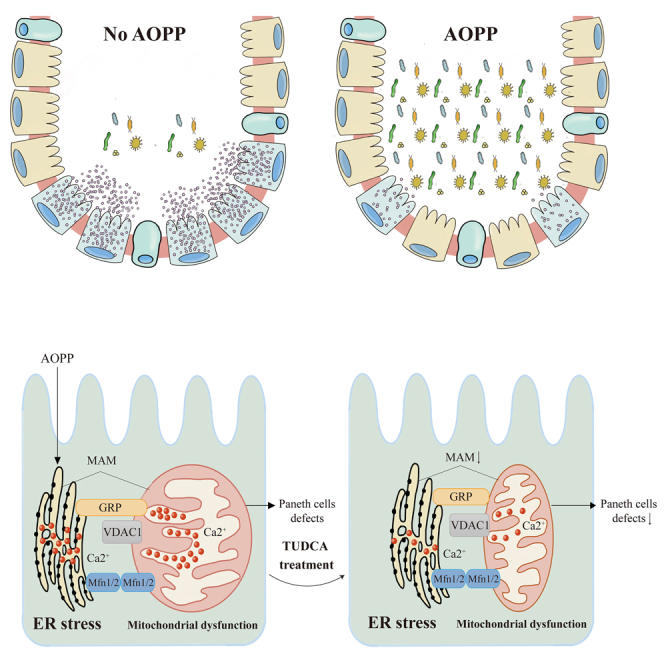

## Introduction

Crohn's disease (CD) is a multifactorial chronic relapsing inflammatory disease of the gastrointestinal tract, exhibiting loss of intestinal epithelial cell (IEC) barrier integrity and dysregulated immune cell responses.^1^ Paneth cells (PC), a kind of special epithelial cells located at the base of crypts, play an important role in regulating intestinal homeostasis by interceding host-microbial relations. PC routinely secrets antimicrobial peptides (lysozyme, cryptdins, and phospholipase A) to protect the host from intestinal pathogenic bacteria and shape the composition of the microflora, which colonizes the small intestine.^2^ PC also functions in maintaining the regeneration of the small intestinal epithelium.^3^ PC defects, represented by the reduced number of PC and impaired production of antimicrobial peptides, contribute to microbiota changes and poor clinical outcomes.^4^^,^^5^^,^^6^ It is regarded that PC defects is a probable hallmark of intestinal inflammation, thus PC-targeted therapeutics may provide a novel strategy for CD.^7^ However, the pathogenic factors and underlying mechanisms driving PC defects remain poorly understood.

It is indicated that endoplasmic reticulum (ER) stress and its associated pathological alternation could play a role in PC defects.^8^ ER stress arises from the insufficiency of ER to deal with protein folding.^9^ To secrete antimicrobial peptides routinely, ER of PC must deal with a very high amount of protein folding, providing more susceptibility to excessive ER stress.^10^^,^^11^ In cases where ER stress cannot be reversed, as intimate organelles of ER, mitochondria-associated ER membranes (MAM) and mitochondria would cooperate to lead to cell death.^10^

In specimens and plasma with active CD, our previous studies confirmed that there was a marked increase of advanced oxidation protein products (AOPP) and further manifested that AOPP could function in the pathogenesis and progression of CD.^12^^,^^13^^,^^14^^,^^15^ Furthermore, Luceri et al. also demonstrated that AOPP may represent a pathogenic factor and a potential therapeutic target of CD.^16^ While AOPP can induce IEC and crypt cell death, it remains unclear whether these protein products can cause PC defects and if the underlying mechanism is ER stress-dependent.^14^ Here, we showed that AOPP-induced PC defects *in vivo* via ER stress could be ameliorated by tauroursodeoxycholic acid (TUDCA), an ER stress inhibitor. These results together indicated that AOPP was involved in triggering PC defects and disrupting intestinal homeostasis during CD.

## Results

### Analysis of AOPP expression associated with PC defects in active CD tissue

**Figure 1:**
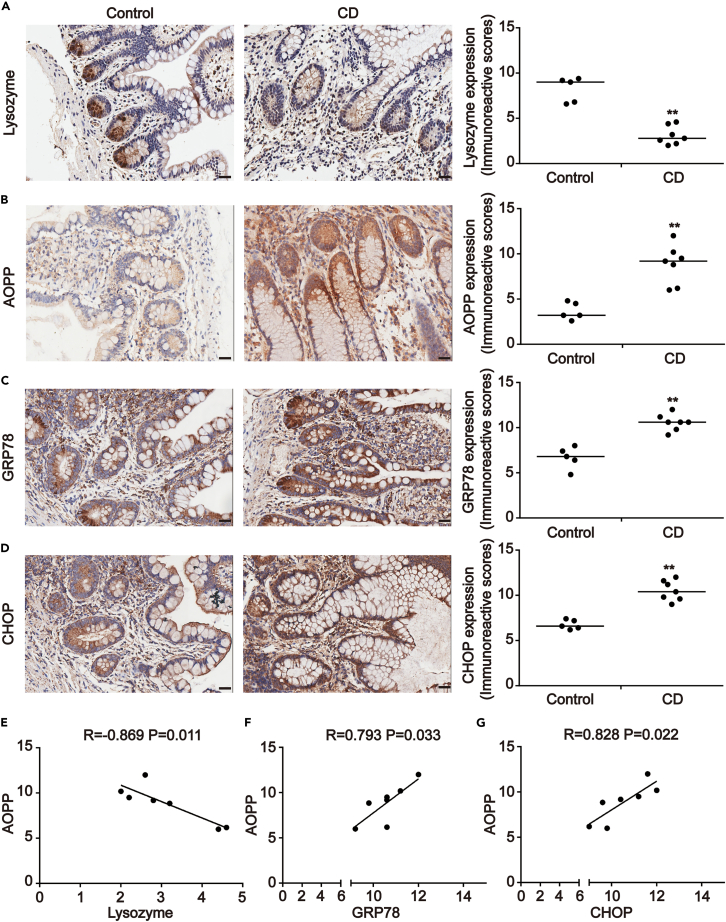
AOPP accumulation correlated with PC and ER stress markers expression in active CD (A–D) Images representative of immunohistochemical staining showed the expression of Lysozyme, AOPP, GRP78 and CHOP, respectively, in intestinal biopsies from 7 patients with CD and 5 healthy controls. Scale bar, 20 μm. Data for the accompanying graphs were generated from immunoreactive scores (IRS). Overall, these results revealed a reduction in Lysozyme (A) with an induction of AOPP (B), GRP78 (C) and CHOP (D). Data were represented by median with range. Student’s *t* test, ∗∗p < 0.01 versus controls. (E) Pearson correlation and linear analysis exhibited that AOPP accumulation was negatively correlated with Lysozyme expression in intestinal crypts of patients with CD (R = −0.869, p = 0.011). (F and G) Pearson correlation and linear analysis uncovered that increased expression of GRP78 and CHOP were positively correlated with AOPP presence in intestinal crypts of patients with CD (R = 0.793, p = 0.033; R = 0.828, p = 0.022). AOPP, advanced oxidation protein products; CD, Crohn disease; ER, endoplasmic reticulum; GRP78, glucose-regulated protein 78; CHOP, CAAT/enhancer-binding protein (C/EBP) homologous protein.

**Figure 2:**
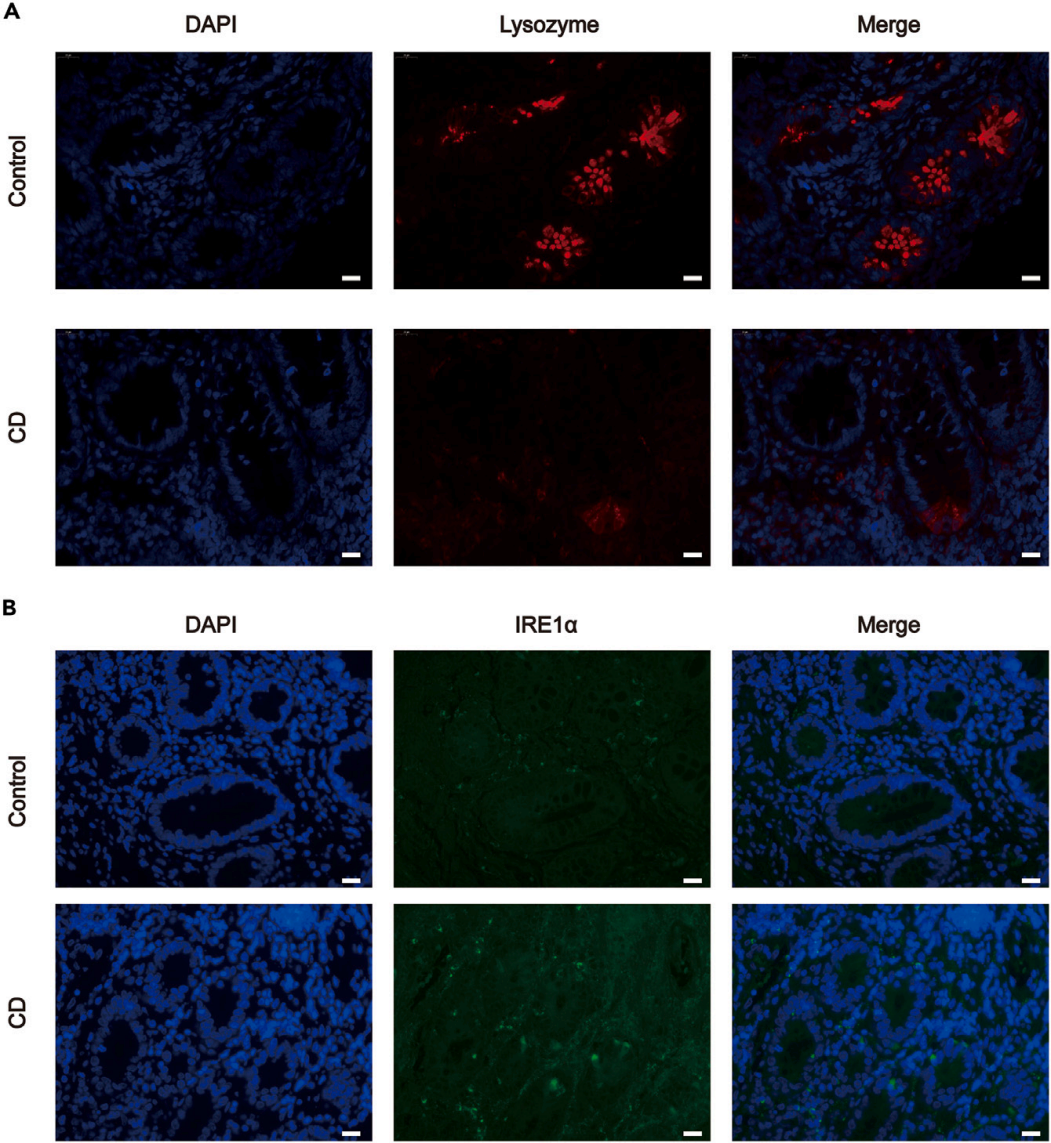
Lysozyme and IRE1α expression in intestinal crypts of patients with CD and controls (A and B) Representative images of immunofluorescence staining for Lysozyme and IRE1α revealed a significant reduction of Lysozyme and enhanced expression of IRE1α in intestinal crypts of patients with CD when compared with healthy intestinal crypts. Scale bar, 20 μm IRE1α, inositol-requiring enzyme 1α.

To investigate the potential role of AOPP in PC defects, AOPP levels were evaluated in biopsy CD tissues. A total of seven CD patients and five healthy subjects were enrolled in this study (Tables 1 and 2).Lysozyme (Lyz) expression was used to assess PC function and identify PC areas in intestinal tissues.^6^^,^^8^^,^^17^ Immunohistochemistry (IHC) staining indicated that Lyz was predominantly located in the intestinal crypts in which PC were located. IHC analysis found that Lyz expression was 2.63-fold lower in CD lesions than in normal tissues (p < 0.01; Figure 1A). Immunofluorescence (IF) revealed that the expression of Lyz was weaker and abnormally distributed in CD tissues, including lower expression in normal secretory granules and diffuse staining in the PC cytoplasm (Figure 2A).

**Table 1:** Clinical characteristics of CD patients (n = 7)

Variables	
Age (years)	30.86 ± 6.23
Gender	
Male	4
Female	3
Smoking	
Never	5
Current	2
Disease state	
Recurrent	4
Newly diagnosed	3
Ongoing therapy	
Biological agents	3
Other therapeutic method	2
Non treatment	2
Laboratory test	
Albumin (g/L)	31.19 ± 5.03
CRP	33.095 ± 23.35
ESR	28.43 ± 13.34

CD, Crohn disease; CRP, C reactive protein; ESR, erythrocyte sedimentation rate.

**Table 2:** Clinical data of healthy controls

Variables	
Age (years)	41.80 ± 5.54
Gender	
Male	1
Female	4
Smoking	
Never	4
Current	1

In accordance with our previous study,^14^ AOPP was mainly detected in inflammatory cells in the lamina propria layer and in the cytoplasm of intestinal crypt cells, where Lyz is also distributed. AOPP immunostaining was nearly 3-fold higher in CD versus healthy intestinal tissue (p < 0.01; Figure 1B). Furthermore, Pearson correlation and linear regression analyses revealed that AOPP accumulation was negatively correlated with Lyz expression in CD tissues (R = −0.869, p = 0.011; Figure 1E). In contrast, AOPP accumulation was not significantly correlated with Lyz expression in healthy controls (Figure S1A). These results suggest that AOPP deposition in intestinal tissues may be involved in the development of PC defects.

### AOPP accumulation correlated with the expression of ER stress markers in active CD tissue

Excessive ER stress contributes to the deterioration of cell function and eventual cell death. This study assessed the presence of ER stress-associated protein markers in the PCs of active CD tissues. ER stress-associated proteins including glucose-regulated protein 78 (GRP78), CAAT/enhancer-binding protein (C/EBP) homologous protein (CHOP), and inositol-requiring enzyme 1α (IRE1α) were detected by IHC or immunofluorescence. Positive immunostaining for GRP78 and CHOP was observed in PC, and the staining intensity was higher in CD lesions than in normal tissues (p < 0.01) (Figures 1C and 1D). The intestinal crypts in active CD tissues also exhibited increased IRE1α staining (Figure 2B).

In our previous study, AOPP deposition correlated with intestinal damage and the epithelial-mesenchymal transition (EMT) in CD, suggesting that AOPP may correlate with the presence of ER stress in active CD crypt epithelial cells.^15^ Statistical analysis was performed to confirm the relationship between AOPP and ER stress markers and the results suggested that AOPP accumulation was positively correlated with GRP78 and CHOP expression in CD intestinal crypts (R = 0.793, p = 0.033; Figure 1F and R = 0.828, p = 0.022; Figure 1G). Meanwhile, AOPP accumulation was not significantly correlated with GRP78 and CHOP expression in healthy controls (Figures S1B and S1C). These findings suggest that there is a correlation between AOPP and ER stress and that the accumulation of AOPP may have functional significance in ER stress-induced PC defects.

### Effect of AOPP challenge on PC defects and ER stress in mice

To investigate whether AOPP affected PC defects by inducing ER stress *in vivo*, normal male C57BL/6 mice were treated with a daily intraperitoneal injection of AOPP for 28 days. The intestinal histopathology and expression of PC and ER stress markers were assessed.

**Figure 3:**
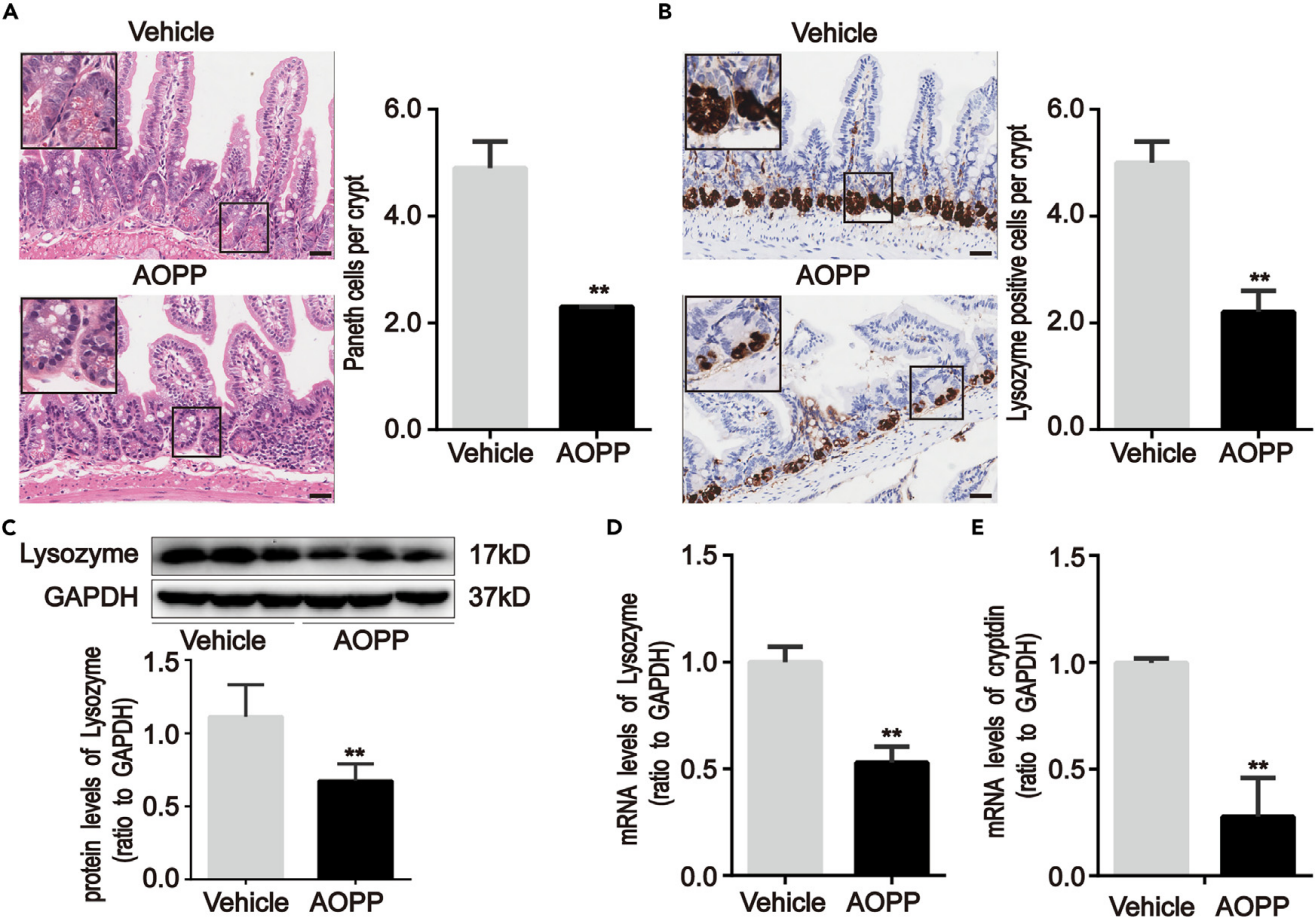
Chronic administration of AOPP induced PC defects in intestinal crypts of mice (A and B) Representative images of HE staining for PC number quantification and immunohistochemical staining for Lysozyme in intestine sections of mice with or without AOPP treatment. These images were representative of results from each experimental group, each with five mice. Images showed that AOPP challenge reduced the PC number and lysozyme expression in intestinal crypts compared with vehicle-treated mice. Scale bar, 20 μm. (C) Representative Western blotting and densitometric quantification showed the protein level of Lysozyme in isolated intestinal crypts of mice with or without AOPP administration. (D and E) Bar graphs representing qPCR analysis showed the mRNA levels of Lysozyme and cryptdins in isolated intestinal crypts of mice with or without AOPP administration. Relative protein and mRNA levels were normalized by GAPDH, respectively. Data were represented by median with range, n = 5 mice; error bars indicating range. Student’s *t* test, ∗∗p < 0.01 versus vehicles. Vehicle, PBS treatment for 28 days; AOPP, AOPP treatment for 28 days; qPCR, quantitative real-time polymerase chain reaction; GAPDH, glyceraldehyde-3-phosphate dehydrogenase.

The AOPP-treated mice showed signs of intestinal inflammation and injury. HE staining revealed that chronic AOPP treatment resulted in a reduction in PC (p < 0.01; Figure 3A). IHC analysis showed that Lyz was widely and strongly expressed in intestinal crypts in vehicle (PBS)-treated mice but had reduced expression in AOPP-treated mice at 28 days (p < 0.01; Figure 3B). AOPP treatment also significantly reduced Lyz protein and mRNA expression in the isolated intestinal crypts of mice (p < 0.01; Figures 3C and 3D) and decreased mRNA expression of crpytdin (also called defensin), an antimicrobial protein produced by PC, suggesting loss of PC function (p < 0.01; Figure 3E).^6^^,^^8^^,^^17^

**Figure 4:**
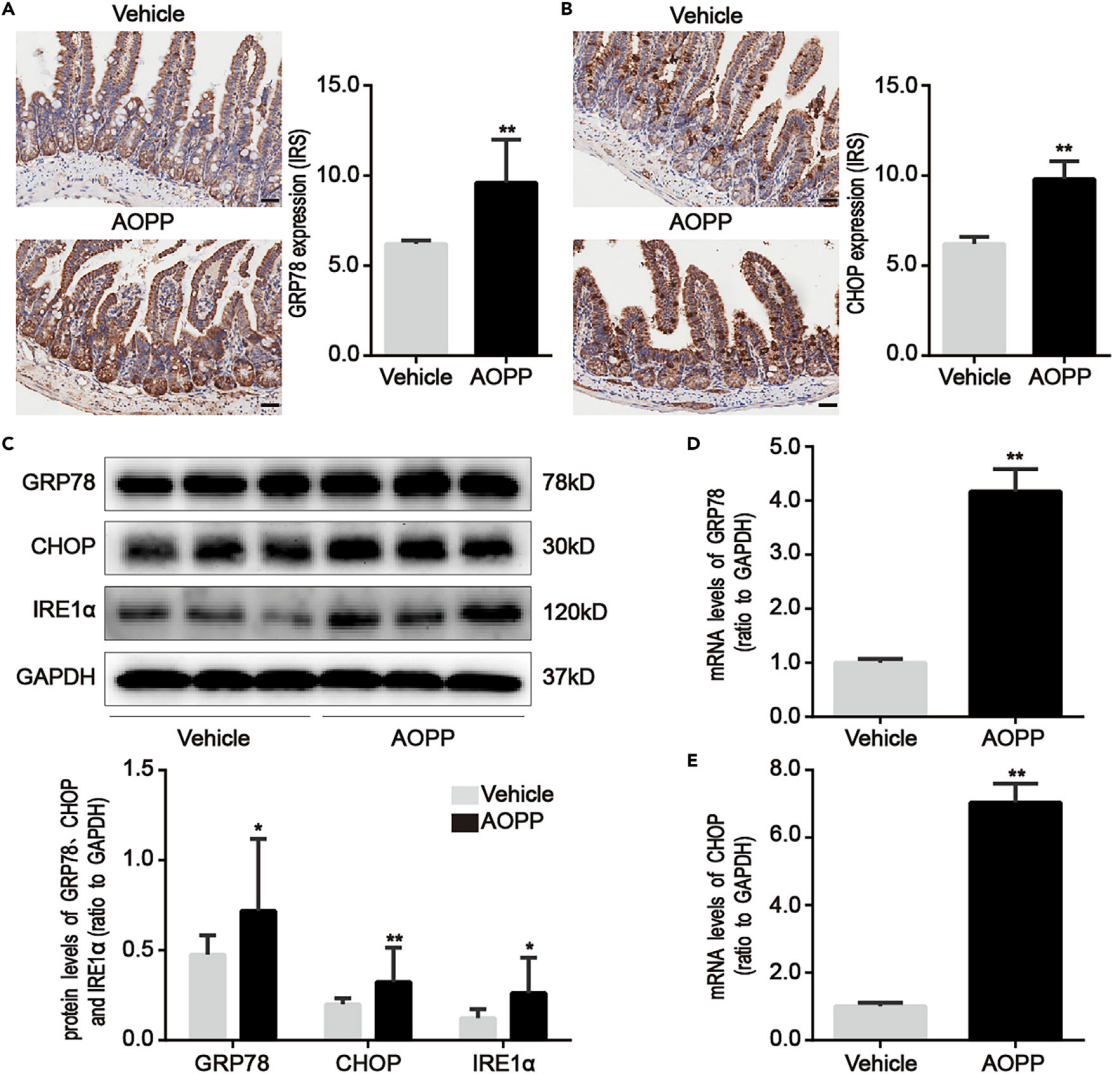
Chronic challenge of AOPP induced ER stress in intestinal crypts of mice (A and B) Representative images of immunohistochemical staining for GRP78 and CHOP in mice with or without AOPP challenge. These images were representative of results from each experimental group, each with five mice. Images showed that AOPP treatment elevated GRP78 and CHOP levels in intestinal crypts compared with vehicle-treated mice. Scale bar, 20 μm. (C) Representative Western blotting and densitometric quantification showed the protein levels of GRP78, CHOP and IRE1α in isolated intestinal crypts of mice with or without AOPP challenge. (D and E) Bar graphs representing qPCR analysis showed the levels of GRP78 and CHOP mRNA in isolated intestinal crypts of mice with or without AOPP challenge. Relative protein and mRNA levels were normalized by GAPDH, respectively. Data were represented by median with range, n = 5 mice; error bars indicating range. Student’s *t* test, ∗p < 0.05 and ∗∗p < 0.01 versus vehicles.

AOPP treatment significantly augmented expression of the ER stress markers, GRP78 and CHOP, in the intestinal crypts of mice (p < 0.01; Figure 4A and 4B). GRP78, CHOP, and IREα protein levels (p < 0.05; Figure 4C) and GRP78 and CHOP mRNA levels (p < 0.01; Figures 4D and 4E) increased in isolated intestinal crypts from AOPP-treated mice. These results suggested that a chronic AOPP challenge could induce PC defects and ER stress.

### TUDCA effectively mitigated AOPP-mediated PC defects

**Figure 5:**
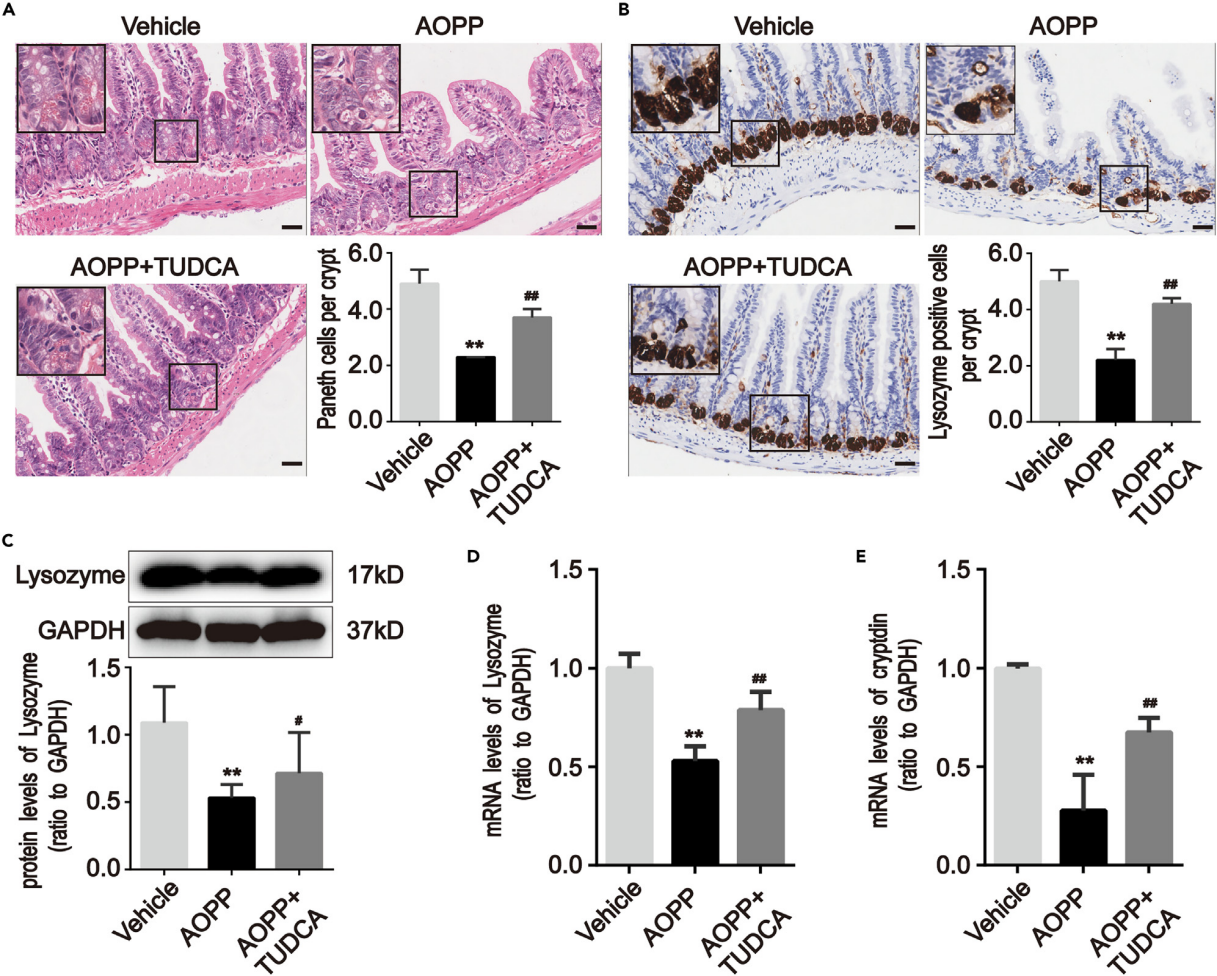
AOPP-mediated PC defects depended on ER stress in intestinal crypts of mice (A and B) Representative images of HE staining for PC number quantification and immunohistochemical staining for Lysozyme in intestine sections of mice that underwent intraperitoneal administration of AOPP with or without TUDCA. These images were representative of results from each experimental group, each with five mice. Images reveled that treatment with TUDCA alleviated AOPP-mediated decreasing PC number and lysozyme expression in intestinal crypts compared with AOPP-treated mice. Scale bar, 20 μm. (C) Representative Western blotting and densitometric quantification revealed the protein level of Lysozyme in isolated intestinal crypts of mice that underwent intraperitoneal administration of AOPP with or without TUDCA. (D and E) Bar graphs representing qPCR analysis revealed the levels of mRNA of Lysozyme and cryptdins in isolated intestinal crypts of mice that underwent intraperitoneal administration of AOPP with or without TUDCA. Relative protein and mRNA levels were normalized by GAPDH, respectively. Data were represented by median with range, n = 5 mice; error bars indicating range. ANOVA, ∗∗p < 0.01 versus vehicles; ^#^p < 0.05 and ^##^p < 0.01 versus AOPP-treated mice.

**Figure 6:**
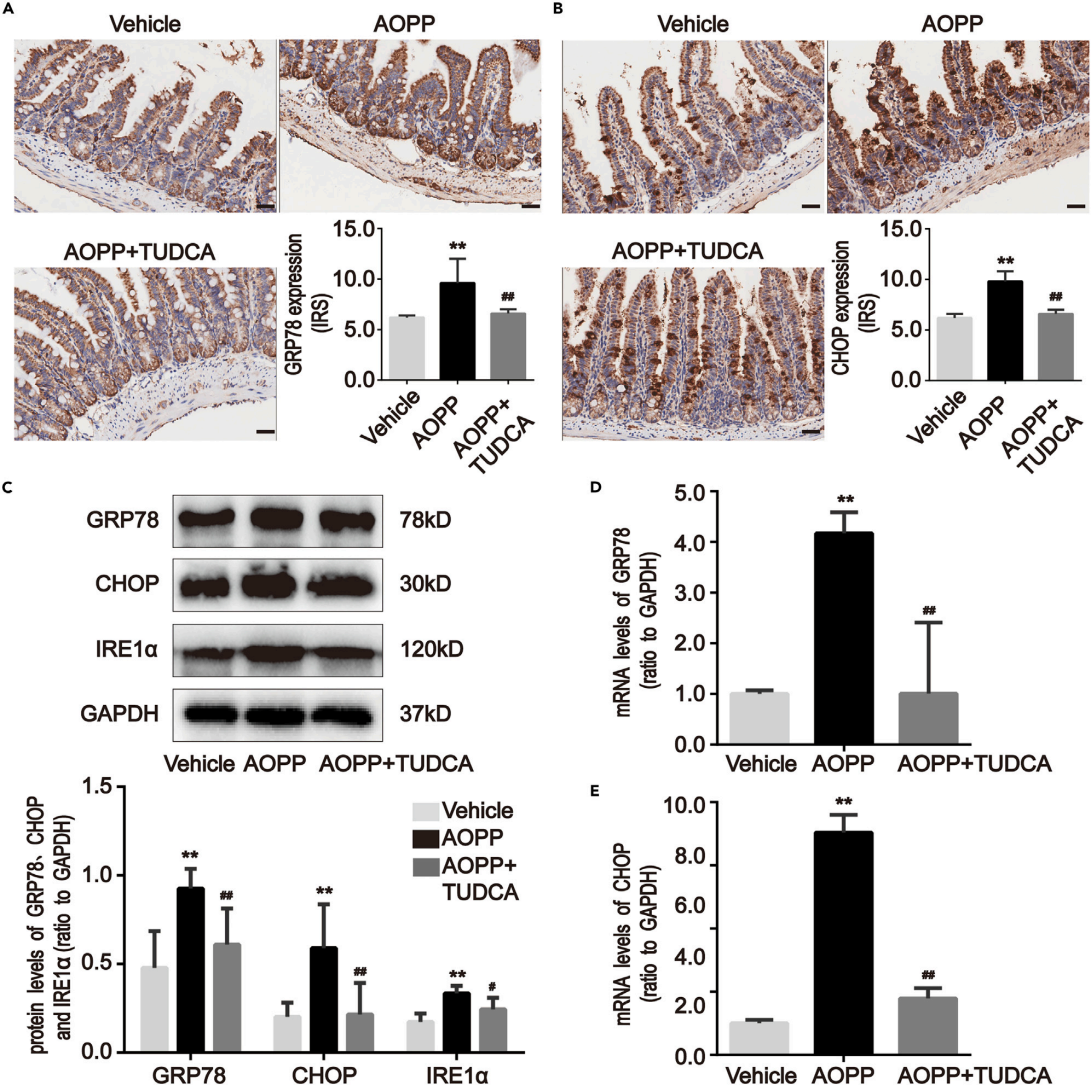
TUDCA inhibited AOPP-resulted ER stress in intestinal crypts of mice (A and B) Representative images of immunohistochemical staining for GRP78 and CHOP in mice that underwent intraperitoneal administration of AOPP with or without TUDCA. These images were representative of results from each experimental group, each with five mice. Images revealed that treatment with TUDCA attenuated AOPP-induced increased GRP78 and CHOP levels in intestinal crypts compared with AOPP-treated mice. Scale bar, 20 μm. (C) Representative Western blotting and densitometric quantification revealed the protein levels of GRP78, CHOP and IRE1α in isolated intestinal crypts of mice that underwent intraperitoneal administration of AOPP with or without TUDCA. (D and E) Bar graphs representing qPCR analysis revealed the levels of GRP78 and CHOP mRNA in isolated intestinal crypts of mice that underwent intraperitoneal administration of AOPP with or without TUDCA. Relative protein and mRNA levels were normalized by GAPDH, respectively. Data were represented by median with range, n = 5 mice; error bars indicating range. ANOVA, ∗∗p < 0.01 versus vehicle; ^#^p < 0.05 and ^##^p < 0.01 versus AOPP-treated mice. AOPP+TUDCA, AOPP treatment plus daily intraperitoneal injection of TUDCA; TUDCA, tauroursodeoxycholic acid, an inhibitor of ER stress.

To confirm the role of ER stress in AOPP-induced PC defects, mice were pretreated with TUDCA, an inhibitor of ER stress, prior to AOPP treatment. TUDCA treatment reversed AOPP-induced reduction in PC numbers in intestinal crypts (p < 0.01; Figure 5A) and increased Lyz expression by PC (p < 0.01; Figure 5B). TUDCA also restored the AOPP-induced reduction of lysozyme and cryptdins in the isolated intestinal crypts (p < 0.05; Figures 5C–5E). To determine the effect of TUDCA on ER stress, the expression of ER stress markers was assessed after TUDCA treatment, and the levels of GRP78, CHOP, and IREα were shown to be significantly reduced (p < 0.05; Figures 6A–6E) These findings suggested that AOPP-induced PC defects are associated with an increase in ER stress.

### TUDCA alleviated AOPP-induced MAM formation and mitochondrial dysfunction

**Figure 7:**
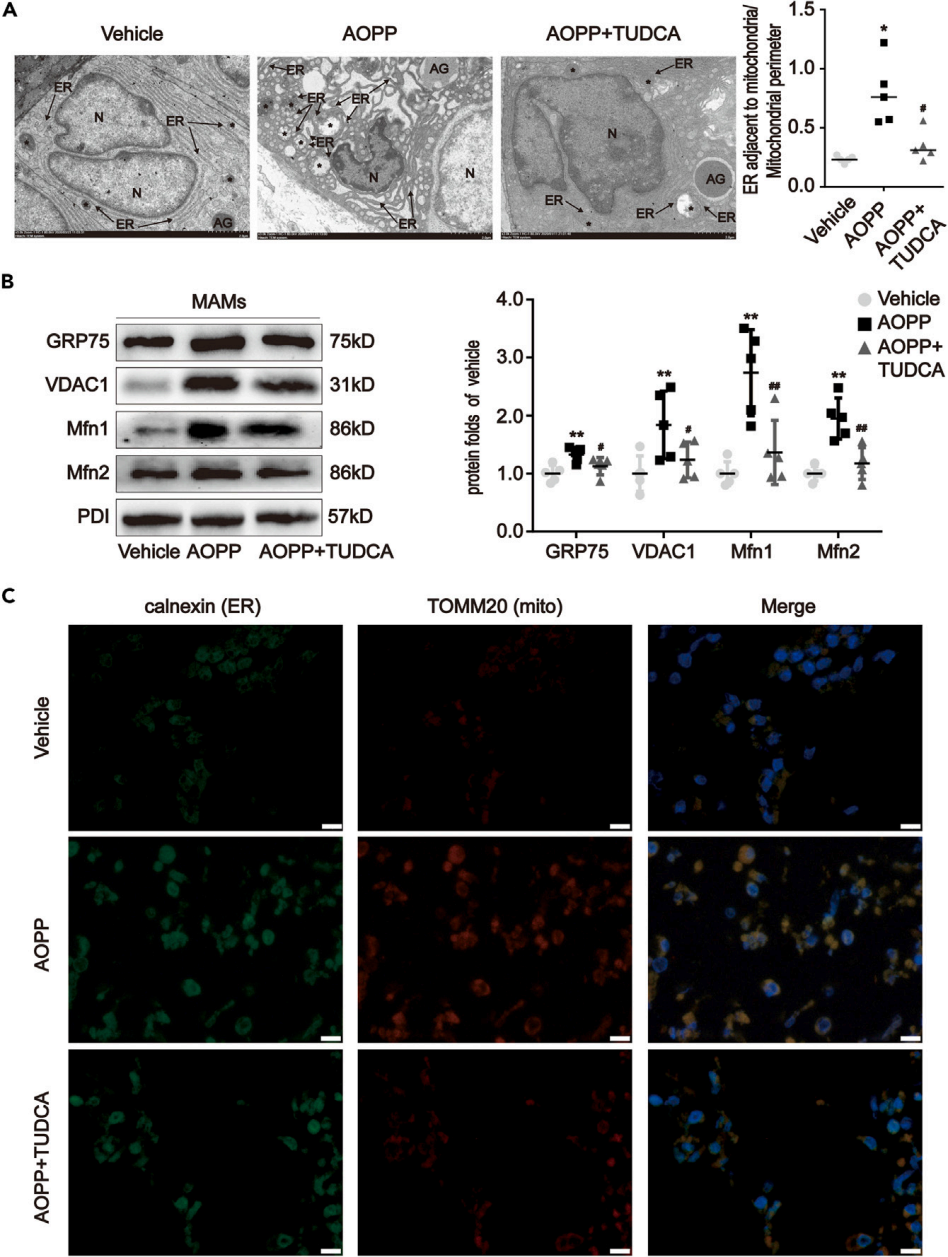
MAM enrichment in AOPP-treated intestinal crypts of mice was mediated by ER stress (A) Representative images of TEM for MAM analysis showed the association of ER with mitochondria in intestinal crypts of mice that underwent intraperitoneal administration of AOPP with or without TUDCA. Quantitation of ER length adjacent to mitochondria normalized by mitochondrial perimeter. Scale bar, 2 μm. (B) Representative Western blotting and densitometric quantification showing that the expression of GRP75, VDAC1, Mfn1, and Mfn2 were enhanced in MAM fractions from isolated intestinal crypts of AOPP-treated mice compared with vehicle-treated mice, and the AOPP+TUDCA group demonstrated that administration with TUDCA restored AOPP-resulted enhancive MAM-related protein. PDI served as a loading control. Relative protein levels were expressed as fold induction over vehicle. Data were represented by median with range, n = 5 mice; error bars indicating range. ANOVA, ∗∗p < 0.01 versus vehicles; ^#^p < 0.05 and ^##^p < 0.01 versus AOPP-treated mice. (C) Representative images of organelle-targeted fluorescence labeling for ER (calnexin) and mitochondria (TOMM20) showed the co-localization of ER and mitochondria, indicating MAM formation in isolated intestinal crypts of mice that underwent intraperitoneal administration of AOPP with or without TUDCA. Scale bar, 10 μm. MAM, mitochondria-associated ER membranes; Grp75, glucose regulated protein 75; VDAC1, voltage-dependent anion channel 1; Mfn1/2, mitofusin-1/2; TEM, transmission electron microscopy; ER, endoplasmic reticulum; Asterisks, mitochondria; N, nucleus; AG, acidophil granule.

Evidence suggests that excessive ER stress can increase cell death by altering MAM and causing mitochondrial dysfunction. To assess this, the morphology of MAM and mitochondria were visualized in the intestinal crypts of control and AOPP-treated mice using transmission electron microscopy. AOPP induced obvious nuclear pyknosis, with increased MAM formation and swollen mitochondria in the intestinal crypts (p < 0.05; Figure 7A). The increased formation of MAM was also demonstrated by Western blotting (p < 0.05; Figure 7B), including higher expression of GRP75, VDAC1, Mfn1, and Mfn2. An increase in AOPP-induced MAM formation was further demonstrated by the colocalization of calnexin and TOMM20 (Figure 7C).

**Figure 8:**
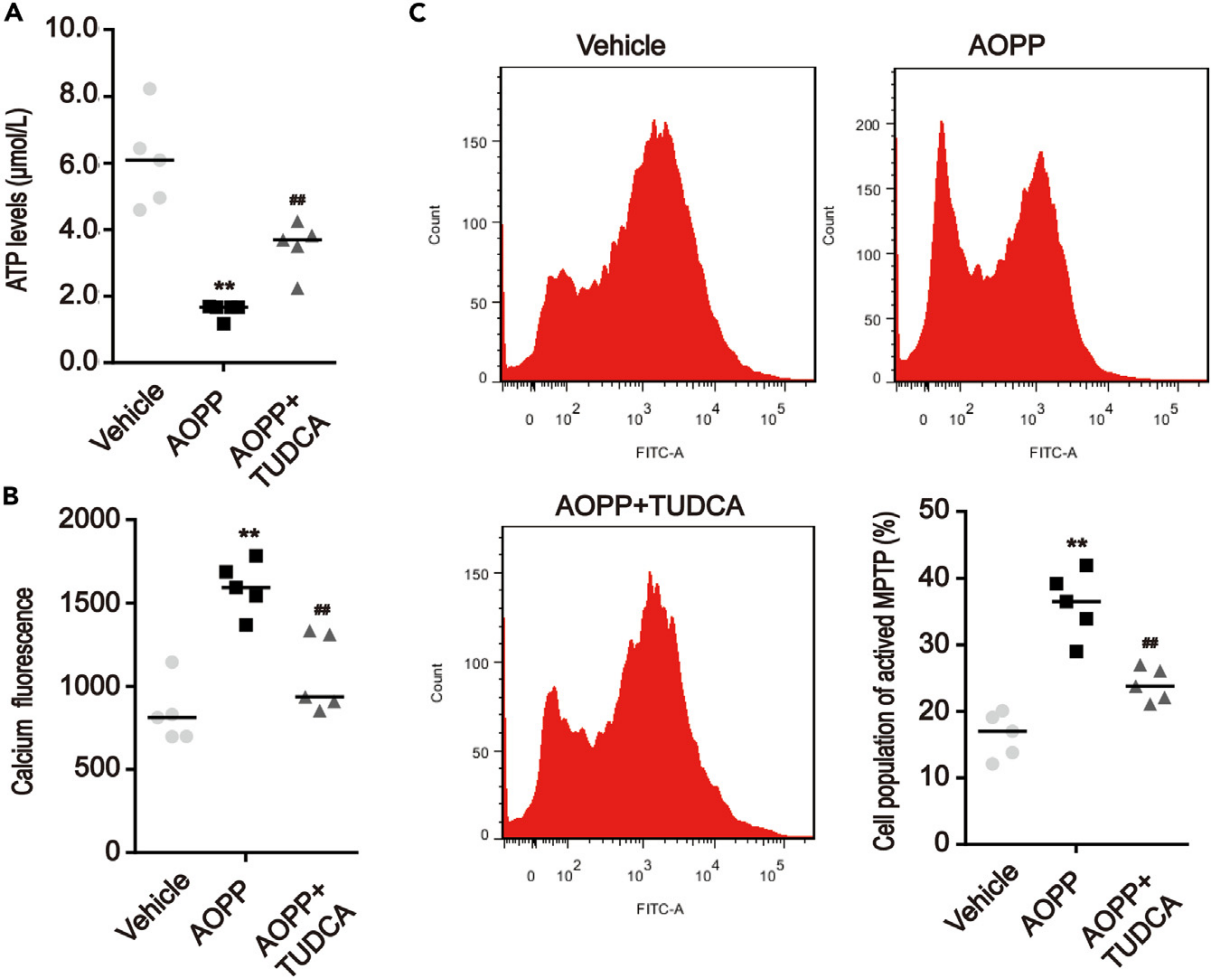
AOPP induced mitochondrial dysfunction in intestinal crypts of mice through ER stress (A) Quantitative luminescence analysis of ATP levels showed the production of ATP in isolated intestinal crypts of mice that underwent intraperitoneal administration of AOPP with or without TUDCA. (B) Quantitative fluorescence analysis of mitochondrial Ca^2+^ levels in isolated intestinal crypts incubating by Rhod-2, a mitochondrial Ca^2+^ indicator, showed that AOPP-treated mice increased levels of mitochondrial Ca^2+^ concentration compared with vehicle-treated mice, and the TUDCA+AOPP group determined that treatment with TUDCA prevented AOPP-induced mitochondrial Ca^2+^ overload. (C) Flow cytometry analysis of MPTP activation showed the opening of MPTP in isolated intestinal crypts of mice that underwent intraperitoneal administration of AOPP with or without TUDCA. Data were represented by median with range, n = 5 mice; error bars indicating range. ANOVA, ∗∗p < 0.01 versus vehicles; ^#^p < 0.05 and ^##^p < 0.01 versus AOPP-treated mice. ATP, adenosine triphosphate; MPTP, mitochondrial permeability transition pore.

ATP production was lower in isolated mitochondria from the intestinal crypts of AOPP-treated mice (p < 0.01; Figure 8A). Mitochondrial Ca^2+^ levels and the opening of MPTP in intestinal crypts were also investigated. By measuring expression of the mitochondrial Ca^2+^ indicator, Rhod-2, quantitative fluorescence analysis revealed a higher mitochondrial Ca^2+^ concentration in the isolated intestinal crypts of AOPP-treated mice than in control mice (p < 0.01; Figure 8B). Flow cytometry showed that the induction of aberrant MPTP opening was also significantly enhanced in AOPP-treated mice (p < 0.01; Figure 8C).

As expected, TUDCA treatment significantly prevented AOPP-induced MAM formation (p < 0.05; Figure 7), mitochondrial swelling, ATP production, mitochondrial Ca^2+^ overloading, and aberrant MPTP opening (p < 0.05; Figure 8). Taken together, these findings indicated that TUDCA was able to reverse AOPP-induced MAM formation and mitochondrial damage.

## Discussion

CD is characterized by excessive damage to the intestinal barrier and abnormal immune responses resulting from commensal bacteria in genetically predisposed individuals.^18^ PC defects contribute to microbiome changes and poor clinical outcomes. In our previous study, aberrant deposition of AOPP in the plasma and intestine was shown to contribute to the pathogenesis and development of CD. These findings suggested that AOPP may be a pathogenic factor and thus, a potential target for CD treatment.^16^ However, the effect of AOPP on PC defects as well as the underlying mechanisms of this relationship has not yet been clarified. The current study found that AOPP and excessive ER stress were associated with PC defects in CD intestinal crypts. AOPP also triggered PC defects *in vivo* which could be ameliorated by TUDCA, an ER stress inhibitor. TUDCA was able to ameliorate AOPP-induced MAM formation and mitochondrial dysfunction. To our knowledge, this is the first study to identify the effects of AOPP on PC, revealing another role for AOPP in the pathogenesis of CD.

PC defects are considered the central element of CD pathogenesis, which causes dysfunction of the microbiome, intestinal epithelial barrier, and immune response.^19^^,^^20^ Our prior studies illustrated that AOPP, a proinflammatory mediator and a novel biomarker of oxidative damage, could induce G1 phase arrest, EMT, and IEC death, thus contributing to CD development and pathogenesis.^13^^,^^14^^,^^15^ The current study showed that AOPP is predominantly colocalized with PC in CD intestinal crypts, and its accumulation is negatively correlated with the expression of Lyz, a secretory granule produced by PC. Thus, it is probable that AOPP may participate in CD development by inducing PC defects. The remarkable secretory activity of PC requires a strong and stable ER to handle large amounts of protein folding.^21^ ER stress is an adaptive reaction that reduces the unfolded protein load to maintain cell viability and function. When this process cannot be reversed, cellular functions deteriorate, often leading to cell death. The current study study found that the deposition of AOPP by PC caused excessive ER stress in active CD tissues.

Identifying the underlying mechanism for AOPP-induced PC defects in CD is required to inform the development of novel methods to treat CD. PC are vulnerable to excessive ER stress. Indeed, ischemia/reperfusion damage and alcohol are also shown to trigger PC defects through ER stress.^22^ AOPP induces cell death through oxidative stress-related signaling and can mediate apoptosis and EMT transition through the reactive oxygen species (ROS)-induced ER stress pathway.^23^^,^^24^^,^^25^^,^^26^^,^^27^ The current study found that AOPP was capable of inducing excessive ROS production, which in turn triggered apoptosis, EMT, and G1 phase arrest in IEC. In addition, the chronic administration of AOPP-induced PC defects in a mouse model by reducing the number of PC, impairing the production of antimicrobial mRNA, including Lyz and crpytidins, by PC, and augmenting the mRNA and protein expression of the ER stress markers, GRP78, and CHOP, in the intestinal crypts. These results together suggested that chronic AOPP challenges could trigger PC defects and ER stress in mice. As a recognized ER stress inhibitor, TUDCA has been effectively used to treat cholestatic liver disease.^28^^,^^29^ TUDCA alleviates intestinal inflammation by suppressing ER stress and was used in a translational phase I study to reduce the symptoms of active ulcerative colitis.^30^ The current study found that TUDCA could effectively reverse AOPP-induced PC defects. TUDCA likely functions by directly inhibiting ER stress.

The maintenance of cellular homeostasis involves the participation of multiple organelles. Increasing evidence suggests that the ER, MAM, and mitochondria can cooperate to cause cellular defects in response to various pathologic stimuli.^22^^,^^31^^,^^32^ ER stress is shown to induce MAM formation which leads to the release of a high level of Ca^2+^ into mitochondria, the aberrant opening of MPTP, and reduced ATP production, eventually causing mitochondrial dysfunction and cellular defects.^33^^,^^34^ The current study found that AOPP-induced MAM formation, high Ca^2+^ release, an MPTP decrease, and mitochondrial dysfunction. These findings indicate that AOPP may induce PC defects by promoting an ER stress-MAM-mitochondrial dysfunction axis. TUDCA was shown to inhibit ER stress and prevent mitochondrial dysfunction to protect PC from the effects of AOPP exposure.

In summary, AOPP may cause PC defects by inducing an ER stress-MAM-mitochondrial dysfunction axis. Targeting AOPP-mediated ER stress-PC defects may thus be a novel approach for treating CD ileitis.

### Limitations of the study

This study provides evidence that AOPP exposure induces PC defects, which may be mediated by ER stress activation. AOPP also caused mitochondria-associated ER membrane formation and mitochondrial dysfunction, all of which were attenuated by administration of the ER stress inhibitor, TUDCA. Considering the multifaceted effects of TUDCA, further study is necessary to elucidate the specific role of ER stress in AOPP-induced PC defects and to comprehensively understand the mechanisms underlying the interaction between TUDCA, ER stress, and PC function.

## STAR★Methods

### Key resources table

**Table:** 

REAGENT or RESOURCE	SOURCE	IDENTIFIER
**Antibodies**		

Mouse AOPP	A gift from Professor Fu Ning, Southern Medical University, Guangzhou, China.^12^^,^^13^^,^^14^^,^^15^	N/A
Rabbit Lysozyme	Abcam	Cat#ab108508
Rabbit GRP78	Proteintech	Cat#11587-1-AP
Rabbit CHOP	Proteintech	Cat#15204-1-AP
Mouse IRE1α	Santa Cruz	Cat# sc-390960
CY3-conjugated Goat anti-Rabbit IgG(H + L)	Servicebio	Cat#GB21303
FITC-conjugated Goat anti-Mouse IgG(H + L)	Servicebio	Cat# GB22301
Rabbit Calnexin	Proteintech	Cat#10427-2-AP
Mouse TOMM20	Santa Cruz	Cat# sc-17764
Mouse GAPDH	Proteintech	Cat# 60004-1-Ig
Rabbit GRP75	Proteintech	Cat#14887-1-AP
Rabbit VDAC1	Proteintech	Cat# 55259-1-AP
Rabbit MFN1	Proteintech	Cat#13798-1-AP
Rabbit MFN2	Proteintech	Cat#12186-1-AP
Rabbit PDI	Proteintech	Cat#11245-1-AP
Mouse GRP78	Santa Cruz	Cat# sc-13539
Mouse CHOP	Santa Cruz	Cat# sc-390310

**Biological samples**		

Intestinal tissues	Nanfang Hospital of Southern Medical University	N/A

**Chemicals, peptides, and recombinant proteins**		

AOPP	Prepared as described previously.^12^^,^^13^^,^^14^^,^^15^	N/A
Rabbit Serum Albumin	Sigma-Aldrich	Cat# 9048-46-8
Sodium hypochlorite	Macklin	Cat#S817439
TUDCA	Selleck	Cat# S3654
EDTA	Guanghua Sci-Tech	Cat#20211120
RIPA lysis buffer	Beyotime	Cat#P0013E
Protease inhibitors	Sigma-Aldrich	Cat#P8215
TRIzol Reagent	Takara	Cat#T9108
PrimeScript™ RT Master Mix	Takara	Cat#RR036A
TB Green™ Premix Ex Taq TM II	Takara	Cat#RR820A
Percoll medium	Sigma-Aldrich	Cat#P7878

**Critical commercial assays**		

Two-step plus Poly-HRP anti-Rabbit IgG Detection System	Zhongsan Jinqiao Biotechnology	Cat# PV-6001
Two-step plus Poly-HRP anti-Mouse IgG Detection System	Zhongsan Jinqiao Biotechnology	Cat# PV-6002
BCA Protein Quantitation Kit	Shenergy Biocolor	Cat#k3000
Cell Mitochondria Isolation Kit	Beyotime	Cat#C3601
ATP Assay Kit	Beyotime	Cat#S0026
BBcellProbeTM CA1 Assay Kit	BestBio	Cat#BB-48122-1
Rhod-2 probe	AAT Bioquest	Cat#145037-81-6

**Experimental models: Organisms/strains**		

Male C57BL/6 mice	Jinan Pengyue Experimental Animal Breeding Center (Shandong, China)	N/A

**Software and algorithms**		

Fiji ImageJ software	NIH – public domain	RRID:SCR_003070
FlowJo v.10.8.1	BD Life Sciences	RRID:SCR_008520
SPSS	IBM	RRID:SCR_002865

### Resource availability

#### Lead contact

Further information and requests for resources and reagents should be directed to and will be fulfilled by the Lead Contact, Fang Xie (stellaff@126.com).

#### Materials availability

This study did not generate new unique reagents.

### Experimental model and study participant details

#### Clinical samples

Intestinal tissues were collected from 7 patients clinically diagnosed with CD and 5 healthy volunteers, who underwent endoscopy at Nanfang Hospital of Southern Medical University (Guangzhou, China) in 2021. The clinical characteristics of patients with CD are summarized in Table 1. All human studies followed the ethical principles of the World Medical Association (WMA) Declaration of Helsinki, and this research was approved by the Medical Ethical Committee of Nanfang hospital (number: NFEC-2021-053), and informed consent was obtained from each patient.

#### Animal

Male C57BL/6 mice (6 weeks, weighing 19–20 g) were obtained from Jinan Pengyue Experimental Animal Breeding Center (Shandong, China) and housed in the Southern Medical University Animal Experiment Center (Guangzhou, China). AOPPs were prepared *in vitro* by mixing Rabbit Serum Albumin (Sigma, St Louis, MO) with sodium hypochlorite (Macklin, Shanghai, China) for 30 min, as described previously.^12^^,^^13^^,^^14^^,^^15^ All mice were randomly divided into three groups (n = 5 in each group) and given intraperitoneal injections of the following: (i) vehicle group: daily injection of phosphate-buffered saline (PBS) for 28 days (pH 7.4); (ii) AOPP group: daily injection of AOPP (50 mg/kg for 28 days); (iii) AOPP plus TUDCA (Selleck, Shanghai, China)-treated group: daily injection of TUDCA (250 mg/kg) for 2 h, before injection of AOPP (50 mg/kg) for 28 days. Mice were anesthetized and exsanguinated after treatment as indicated, and the entire intestinal tissues were removed for further investigation. The experimental procedures with animals complied with ARRIVE guidelines and were performed in accordance with the U.K. Animals (Scientific Procedures) Act, 1986. In addition, all animal experiments were approved by the Laboratory Animal Care and Use Committee of Southern Medical University (Number: L2017138).

### Method details

#### Intestinal crypts isolation

After Peyer’s patch being removed, the collected intestinal tissues were flushed with cold PBS, and opened longitudinally. Mucus and villi were removed by gently scraping with a glass side. Intestines were then washed 3 times in cold PBS, chopped into small pieces, and incubated in cold PBS containing 2 mM EDTA (Guanghua Sci-Tech, Guangdong, China) for 30 min at 4°C with gentle agitation. After vigorous shaking, the supernatant was discarded using a 70 μm strainer, and the intestinal pieces were transferred to another 2 mM EDTA in PBS for 15 min at 4°C with gentle agitation, then the supernatant was collected. The intestinal pieces were resuspended in 2 mM EDTA for another round of incubation. After 10 min, the intestinal pieces were discarded, and crypts were strained by centrifugation of both collected supernatant at 600g for 5 min. Crypts were then resuspended in cold PBS for further analysis.

#### Immunohistochemistry, immunofluorescence, H&E staining and PC counting

Paraffin-embedded intestinal tissue sections were deparaffinized in xylene, rehydrated in graded alcohols, retrieved in citrate buffer, and blocked in serum. Immunohistochemistry was performed using primary antibodies against AOPP (A gift from Professor Fu Ning, Southern Medical University, Guangzhou, China), Lysozyme (Abcam), GRP78 and CHOP (Proteintech, Wuhan, China) and secondary antibodies involved in Two-step plus Poly-HRP anti-Rabbit/Mouse IgG Detection System (Zhongsan Jinqiao Biotechnology, Beijing, China), per standard protocols. Immunoreactive scores (IRS) were obtained from multiplication of the positive percentage and intensity of staining. The final score was calculated for each sample. Samples were classified according to the percentage of positive cells: 0 (0% of labeled cells), 1 (<25%), 2 (26–50%), 3 (51–75%) and 4 (>76% of labeled cells); and staining intensity: 0, negative; 1, weak signal; 2, moderate signal; and 3, strong signal. Immunofluorescence was performed using anti-Lysozyme primary (Abcam)/CY3-conjugated Goat anti-Rabbit IgG(H + L) for Lysozyme and anti-IRE1α primary (Santa Cruz)/FITC-conjugated Goat anti-Mouce IgG(H + L) (Servicebio, Wuhan, China) for IRE1α using standard protocols. Additionally, agar-embedded isolated intestinal crypts of mice were co-incubated with primary antibodies against calnexin (Proteintech, Wuhan, China) and TOMM20 (Santa Cruz) to assess co-localization of ER and mitochondria. Hematoxylin and eosin (H&E) staining was performed according to standard protocols, and the quantification of PC was evaluated in a blinded fashion by recording PC numbers in 20 crypts from each mouse. All slides were examined with an Olympus BX-53 microscope (Olympus).

#### Western blotting analysis

Isolated intestinal crypts and MAM fractions of mice were homogenized in RIPA lysis buffer (Beyotime, Shanghai, China) containing protease inhibitors (Sigma), and protein contents were measured using the BCA protein quantitation Kit (Shenergy Biocolor, Shanghai, China). Lysates were then subjected to Western blotting with specific antibodies according to published protocols.^13^ The following primary antibodies were used. Anti-rabbit Lysozyme was purchased from Abcam. Anti-mouse GAPDH, anti-rabbit GRP75, anti-rabbit VDAC1, anti-rabbit Mfn1/2 and anti-rabbit PDI were all purchased from Proteintech (Wuhan, China). Anti-mouse GRP78, anti-mouse CHOP, and anti-mouse IRE1α were all purchased from Santa Cruz.

#### Quantitative real-time PCR (RT-qPCR)

Total RNA was prepared from isolated intestinal crypts of mice using TRIzol reagent (Takara, Dalian, China) in accordance with the manufacturer’s protocols. Complementary DNA was transcribed using PrimeScript RT Master Mix (Takara) and RT-qPCR was performed using TB Green Premix Ex Taq TM II (Takara). Reactions were performed using the LightCycler 480 System (Roche, Basel, Switzerland) according to previous description. The primer sequences of Lysozyme, cryptdins, GRP78 and CHOP are listed in table. Results were normalized to glyceraldehyde-3-phosphate dehydrogenase (GAPDH) and were calculated using the 2-ΔΔCT method.

**Table:** Primer sequences

Primers	5′-3′
GAPDH-Mus Lysozyme-Mus Cryptdins-Mus GRP78-Mus CHOP-Mus	TGTGTCCGTCGTGGATCTGA TTGCTGTTGAAGTCGCAGGAG GCCAAGGTCTACAATCGTTGTGAGTTG CAGTCAGCCAGCTTGACACCACG AAGAGACTAAAACTGAGGAGCAGC GGTGATCATCAGACCCCAGCATCAGT ATCAGCAAACTCTATGGAAGTGGA TCTCTCAATTTTCTCCCAACGA GAGCTGGAAGCCTGGTATGAG CAGGGTCAAGAGTAGTGAAGGTTTT

#### MAM fractionation

MAM fractions were isolated from intestinal crypts of mice based on published protocols.^35^ Briefly, 1.0 × 10 ^9^/mL isolated crypts were washed twice with cold PBS in a 50 mL polypropylene tube and centrifuged at 600g for 5 min at 4°C. The supernatant was discarded. The pellet was resuspended in 20 mL of cold IBcells–1 (225 mM mannitol, 75 mM sucrose, 0.1 mM EGTA and 30 mM Tris–HCl pH 7.4). and then was disrupted by dounce homogenization. The homogenate was spun at 600g for 5 min at 4°C; the supernatant was collected and further centrifuged at 7,000g for 10 min at 4°C. The pellet containing mitochondria was gently resuspended in 20 mL of cold IBcells–2 (225 mM mannitol, 75 mM sucrose and 30 mM Tris–HCl pH 7.4) and purified further by centrifuging twice at 7,000g and 10,000g for 10 min at 4°C respectively. The obtained crude mitochondrial pellet was resuspended gently in 2 mL of cold MRB buffer (250 mM mannitol, 5 mM HEPES (pH 7.4) and 0.5 mM EGTA) and purified by centrifugation at 95,000g for 30 min on a Percoll medium (Sigma-Aldrich). MAM was identified as the diffused white band located above the mitochondrial fraction on the Percoll medium and obtained by differential centrifugation as described. Protein concentration was measured using the BCA Protein Quantitation Kit (Shenergy Biocolor) and 25 μg of protein was analyzed by Western blotting as indicated above.

#### Transmission electron microscopy

The intestinal tissues of mice were fixed with 2.5% glutaraldehyde for 1 h at room temperature and 3 h at 4°C, then replaced the glutaraldehyde with PBS. After dehydration in a graded series of alcohol (15 min each; 50%, 70%, 80%, 90%, 95%, 100% × 2), the tissues were embedded in Resin mixture and generated 70 nm ultrathin sections using a glass knife on a Leica Ultracut R cutter. The sections were stained with uranyl acetate and lead citrate for 15 min each，and examined in a Hitachi HT-7700 transmission electron microscopy (TEM; Hitachi High-Technologies Europe GmbH). The ER mitochondrial contacts were quantified as described previously.^36^ Briefly, the images were analyzed using ImageJ. The mitochondrial and ER membranes were delineated using the freehand tool. The selected areas were converted to masks and the total number, the perimeter of ER and mitochondria were calculated. Two independent investigators quantified the images blindly. For the acquisition of MAM quantification, we normalized the total ER connected to mitochondria to total mitochondrial perimeter.

#### Mitochondria fractionation from mice intestinal crypts

Mitochondrial fractions of intestinal crypts from mice were prepared using the Cell Mitochondria Isolation Kit (Beyotime Institute of Biotechnology) by the manufacturer’s protocol. Briefly, intestinal crypts were collected and washed twice with cold PBS and resuspended with Lysis buffer. After vortex and incubation on ice for 15 min, the pellets were homogenized for 25 St and the homogenates were centrifuged at 650 g for 10 min at 4°C. The supernatants were centrifuged at 11,000g for 15 min at 4°C. The resulting pellets were mitochondria fractionations.

#### Detection of adenosine triphosphate (ATP) levels

ATP levels in mitochondria of intestinal crypts from mice were measured using an ATP Assay Kit (Beyotime, Shanghai, China) according to the manufacturer’s protocols. The isolated mitochondria were lysed and centrifuged at 12,000g for 5 min at 4°C. After mixing the supernatants (100 μL) with ATP detection working solution (100 μL), the luminescence produced was measured with a luminometer counter (Molecular Devices, Shanghai, China), and the concentration of ATP was calculated using an ATP standard curve.

#### Mitochondrial Ca^2+^ measurement

For the functional study, isolated intestinal crypts were then resuspended in cold PBS. After that, mitochondrial Ca^2+^ levels in intestinal crypts of mice were measured with Rhod-2 probe (AAT Bioquest, Inc.) according to the manufacturer’s instructions. We added 100 μL of the dye-working solution into the wells already containing 100 μL of culture medium and incubated the dye-loading plate at room temperature for 30 min. Then replaced the dye working solution with the HHBS buffer with 1.0 mM Probenecid. Finally, put the plate into a fluorescence microplate reader (Molecular Devices, Shanghai, China) and ran the experiment as Ex/Em = 549/578 nm to collect Rhod-2 signals.

#### Assays of mitochondrial permeability transition pore

For the functional study, single-cell suspension of intestinal crypts was obtained. Mitochondrial permeability transition pore (MPTP) in intestinal crypts of mice was measured using a BBcellProbeTM CA1 Assay Kit (BestBio, Shanghai, China) in accordance with the manufacturer’s instructions. Prepared isolated intestinal crypts (1 × 10 ^6^/mL) and divided the crypts into three parts (500 μL per tube), and recorded them. Added 3 μL of BBcellProbeTM CA1 probe working solution into the tubes respectively. Next, added 5 μL of quenching agent into two of the tubes, and added 5 μL of permeable agent into one of the tubes. After incubating in the dark at 37°C for 15 min, added 3 mL of 1x MPTP dye buffer into the tubes respectively and centrifuged to collect crypts. The crypts were resuspended in cold PBS and collected by flow cytometry (BD FACSCalubur, USA). Quantitative analysis of flow cytometry data was determined using FlowJo (v10) software.

### Quantification and statistical analysis

Values were expressed as the median and range and all experiments were repeated at least in triplicate. Statistical analyses were performed using SPSS 20.0 software (SPSS, Inc., Chicago, IL). The Student’s *t* test was used to assess the differences between two groups. Pearson correlation and linear regression analyses were used to determine the correlation between AOPP and Lysozyme, GRP78, or CHOP. To compare treatment with vehicle, AOPP alone, and TUDCA + AOPP, a one-way analysis of variance (ANOVA) and pairwise comparisons were conducted and evaluated using the LSD or Dunnett’s T3 method, as appropriate. Significance was set at p < 0.05.

## Data Availability

• Data reported in this paper will be shared by the lead contact upon request. • This paper does not report original code. • Any additional information required to reanalyze the data reported in this paper is available from the lead contact upon request.

## References

[1] Torres J., Mehandru S., Colombel J.F., Peyrin-Biroulet L. (2017). Crohn’s disease. Lancet.

[2] Yu S., Balasubramanian I., Laubitz D., Tong K., Bandyopadhyay S., Lin X., Flores J., Singh R., Liu Y., Macazana C. (2020). Paneth Cell-Derived Lysozyme Defines the Composition of Mucolytic Microbiota and the Inflammatory Tone of the Intestine. Immunity.

[3] Clevers H.C., Bevins C.L. (2013). Paneth cells: maestros of the small intestinal crypts. Annu. Rev. Physiol..

[4] Deuring J.J., Fuhler G.M., Konstantinov S.R., Peppelenbosch M.P., Kuipers E.J., de Haar C., van der Woude C.J. (2014). Genomic ATG16L1 risk allele-restricted Paneth cell ER stress in quiescent Crohn’s disease. Gut.

[5] Jackson D.N., Panopoulos M., Neumann W.L., Turner K., Cantarel B.L., Thompson-Snipes L., Dassopoulos T., Feagins L.A., Souza R.F., Mills J.C. (2020). Mitochondrial dysfunction during loss of prohibitin 1 triggers Paneth cell defects and ileitis. Gut.

[6] VanDussen K.L., Liu T.-C., Li D., Towfic F., Modiano N., Winter R., Haritunians T., Taylor K.D., Dhall D., Targan S.R. (2014). Genetic variants synthesize to produce paneth cell phenotypes that define subtypes of Crohn’s disease. Gastroenterology.

[7] Khaloian S., Rath E., Hammoudi N., Gleisinger E., Blutke A., Giesbertz P., Berger E., Metwaly A., Waldschmitt N., Allez M., Haller D. (2020). Mitochondrial impairment drives intestinal stem cell transition into dysfunctional Paneth cells predicting Crohn’s disease recurrence. Gut.

[8] Adolph T.E., Tomczak M.F., Niederreiter L., Ko H.-J., Böck J., Martinez-Naves E., Glickman J.N., Tschurtschenthaler M., Hartwig J., Hosomi S. (2013). Paneth cells as a site of origin for intestinal inflammation. Nature.

[9] Wiseman R.L., Mesgarzadeh J.S., Hendershot L.M. (2022). Reshaping endoplasmic reticulum quality control through the unfolded protein response. Mol. Cell..

[10] Csordás G., Weaver D., Hajnóczky G. (2018). Endoplasmic Reticulum-Mitochondrial Contactology: Structure and Signaling Functions. Trends Cell Biol..

[11] Kaser A., Lee A.-H., Franke A., Glickman J.N., Zeissig S., Tilg H., Nieuwenhuis E.E.S., Higgins D.E., Schreiber S., Glimcher L.H., Blumberg R.S. (2008). XBP1 links ER stress to intestinal inflammation and confers genetic risk for human inflammatory bowel disease. Cell.

[12] Liao Y., Xu J., Qin B., Shi J., Qin C., Xie F., Ou S., Tang J., Wang W., Wu F., Bai L. (2021). Advanced oxidation protein products impair autophagic flux in macrophage by inducing lysosomal dysfunction via activation of PI3K-Akt-mTOR pathway in Crohn’s disease. Free Radic. Biol. Med..

[13] Shi J., Sun S., Liao Y., Tang J., Xu X., Qin B., Qin C., Peng L., Luo M., Bai L., Xie F. (2019). Advanced oxidation protein products induce G1 phase arrest in intestinal epithelial cells via a RAGE/CD36-JNK-p27kip1 mediated pathway. Redox Biol..

[14] Xie F., Sun S., Xu A., Zheng S., Xue M., Wu P., Zeng J.H., Bai L. (2014). Advanced oxidation protein products induce intestine epithelial cell death through a redox-dependent, c-jun N-terminal kinase and poly (ADP-ribose) polymerase-1-mediated pathway. Cell Death Dis..

[15] Xu X., Sun S., Xie F., Ma J., Tang J., He S., Bai L. (2017). Advanced Oxidation Protein Products Induce Epithelial-Mesenchymal Transition of Intestinal Epithelial Cells via a PKC δ-Mediated, Redox-Dependent Signaling Pathway. Antioxidants Redox Signal..

[16] Luceri C., Bigagli E., Agostiniani S., Giudici F., Zambonin D., Scaringi S., Ficari F., Lodovici M., Malentacchi C. (2019). Analysis of Oxidative Stress-Related Markers in Crohn’s Disease Patients at Surgery and Correlations with Clinical Findings. Antioxidants.

[17] Cadwell K., Liu J.Y., Brown S.L., Miyoshi H., Loh J., Lennerz J.K., Kishi C., Kc W., Carrero J.A., Hunt S. (2008). A key role for autophagy and the autophagy gene Atg16l1 in mouse and human intestinal Paneth cells. Nature.

[18] Roda G., Chien Ng S., Kotze P.G., Argollo M., Panaccione R., Spinelli A., Kaser A., Peyrin-Biroulet L., Danese S. (2020). Crohn’s disease. Nat. Rev. Dis. Prim..

[19] Wehkamp J., Stange E.F. (2020). An Update Review on the Paneth Cell as Key to Ileal Crohn’s Disease. Front. Immunol..

[20] Yang E., Shen J. (2021). The roles and functions of Paneth cells in Crohn’s disease: A critical review. Cell Prolif..

[21] Hosomi S., Kaser A., Blumberg R.S. (2015). Role of endoplasmic reticulum stress and autophagy as interlinking pathways in the pathogenesis of inflammatory bowel disease. Curr. Opin. Gastroenterol..

[22] Gyongyosi B., Cho Y., Lowe P., Calenda C.D., Iracheta-Vellve A., Satishchandran A., Ambade A., Szabo G. (2019). Alcohol-induced IL-17A production in Paneth cells amplifies endoplasmic reticulum stress, apoptosis, and inflammasome-IL-18 activation in the proximal small intestine in mice. Mucosal Immunol..

[23] Valente A.J., Yoshida T., Clark R.A., Delafontaine P., Siebenlist U., Chandrasekar B. (2013). Advanced oxidation protein products induce cardiomyocyte death via Nox2/Rac1/superoxide-dependent TRAF3IP2/JNK signaling. Free Radic. Biol. Med..

[24] Zhou L.L., Hou F.F., Wang G.B., Yang F., Xie D., Wang Y.P., Tian J.W. (2009). Accumulation of advanced oxidation protein products induces podocyte apoptosis and deletion through NADPH-dependent mechanisms. Kidney Int..

[25] Liang X., Duan N., Wang Y., Shu S., Xiang X., Guo T., Yang L., Zhang S., Tang X., Zhang J. (2016). Advanced oxidation protein products induce endothelial-to-mesenchymal transition in human renal glomerular endothelial cells through induction of endoplasmic reticulum stress. J. Diabet. Complicat..

[26] Rong G., Tang X., Guo T., Duan N., Wang Y., Yang L., Zhang J., Liang X. (2015). Advanced oxidation protein products induce apoptosis in podocytes through induction of endoplasmic reticulum stress. J. Physiol. Biochem..

[27] Ye W., Zhu S., Liao C., Xiao J., Wu Q., Lin Z., Chen J. (2017). Advanced oxidation protein products induce apoptosis of human chondrocyte through reactive oxygen species-mediated mitochondrial dysfunction and endoplasmic reticulum stress pathways. Fundam. Clin. Pharmacol..

[28] Wang W., Zhao J., Gui W., Sun D., Dai H., Xiao L., Chu H., Du F., Zhu Q., Schnabl B. (2018). Tauroursodeoxycholic acid inhibits intestinal inflammation and barrier disruption in mice with non-alcoholic fatty liver disease. Br. J. Pharmacol..

[29] Paumgartner G., Beuers U. (2002). Ursodeoxycholic acid in cholestatic liver disease: mechanisms of action and therapeutic use revisited. Hepatology.

[30] Li P., Fu D., Sheng Q., Yu S., Bao X., Lv Z. (2019). TUDCA attenuates intestinal injury and inhibits endoplasmic reticulum stress-mediated intestinal cell apoptosis in necrotizing enterocolitis. Int. Immunopharm..

[31] Giorgi C., Missiroli S., Patergnani S., Duszynski J., Wieckowski M.R., Pinton P. (2015). Mitochondria-associated membranes: composition, molecular mechanisms, and physiopathological implications. Antioxidants Redox Signal..

[32] Missiroli S., Patergnani S., Caroccia N., Pedriali G., Perrone M., Previati M., Wieckowski M.R., Giorgi C. (2018). Mitochondria-associated membranes (MAMs) and inflammation. Cell Death Dis..

[33] Orrenius S., Zhivotovsky B., Nicotera P. (2003). Regulation of cell death: the calcium-apoptosis link. Nat. Rev. Mol. Cell Biol..

[34] Rizzuto R., De Stefani D., Raffaello A., Mammucari C. (2012). Mitochondria as sensors and regulators of calcium signalling. Nat. Rev. Mol. Cell Biol..

[35] Wieckowski M.R., Giorgi C., Lebiedzinska M., Duszynski J., Pinton P. (2009). Isolation of mitochondria-associated membranes and mitochondria from animal tissues and cells. Nat. Protoc..

[36] Arruda A.P., Pers B.M., Parlakgül G., Güney E., Inouye K., Hotamisligil G.S. (2014). Chronic enrichment of hepatic endoplasmic reticulum-mitochondria contact leads to mitochondrial dysfunction in obesity. Nat. Med..

