# Supplementary material for: Advanced oxidation protein products induce Paneth cells defects by endoplasmic reticulum stress in Crohn's disease
Source: iScience. 2023 Jul 10;26(8):107312. doi: 10.1016/j.isci.2023.107312 (PMC10393771; doi:10.1016/j.isci.2023.107312)
Supplement: Document S1. Figure S1 [file mmc1.pdf]

iScience, Volume *26*

Supplemental information

Advanced oxidation protein products induce Paneth cells defects by endoplasmic reticulum stress in Crohn's disease

Jie Shi, Weidong Wang, Shibo Sun, Xiaoping Xu, Jieying Fei, Qian Zhou, Caolitao Qin, Shiyu Ou, Fengfei Wu, Fang ting Wu, Tianyan Xu, Lan Bai, and Fang Xie

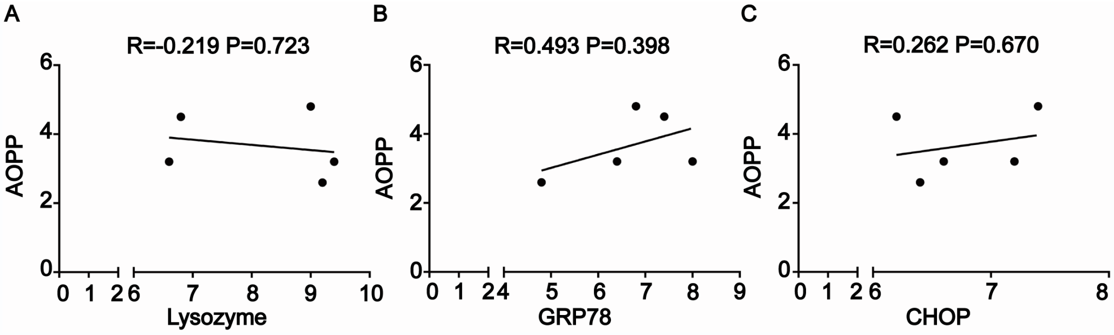

Supplementary Fig. 1. AOPP accumulation was insignificantly correlated with PCs and ER stress markers expression in healthy controls, related to Figure 1. (A-C) Pearson correlation and linear analysis exhibited that AOPP accumulation was insignificantly correlated with expression of Lysozyme (R=-0.219, p=0.723), GRP78 (R=0.493, p=0.398), and CHOP (R=0.262, p=0.670) in healthy controls. AOPP, advanced oxidation protein products; GRP78, glucose-regulated protein 78; CHOP, CAAT/enhancer-binding protein (C/EBP) homologous protein.
